# Comparing Melanoma Invasiveness in Dermatologist- versus Patient-Detected Lesions: A Retrospective Chart Review

**DOI:** 10.1155/2012/187963

**Published:** 2012-07-16

**Authors:** Cindy L. Lamerson, Kristina Eaton, Joel L. Sax, Mohammed Kashani-Sabet

**Affiliations:** ^1^University of Nevada School of Medicine, Reno, NV 89557, USA; ^2^Nevada Center for Dermatology, 650 Sierra Rose Drive, Suite A, Reno, NV 89511, USA; ^3^University of California, Irvine, CA 92868, USA; ^4^Center for Melanoma Research and Treatment, California Pacific Medical Center and CPMC Research Institute, San Francisco, CA 94143, USA

## Abstract

This study examined whether patient-identified melanomas were more advanced than dermatologist-identified tumors at routine clinic visits, and whether a personal or family history of skin cancer was associated with patterns of detection. A retrospective chart review was performed on melanoma patients (*N* = 201) in a private dermatology clinic. Variables included age, gender, pattern of detection (i.e., patient or a board certified dermatologist), personal or family history of skin cancer, skin type, and previous sun exposure, as well as tumor location and severity. Dermatologist-diagnosed melanomas were less invasive (*P* < 0.0005), and more likely present on the chest, back, and legs (*P* < 0.01). Conversely, patient-identified lesions were more likely to occur on the face, neck and scalp, be associated with younger patients, and a family history of melanoma, but not other types of skin cancer (*P* < 0.01). In a post-hoc analysis examining these factors as predictors of tumor invasiveness, only diagnostic source was significant. Specifically, dermatologist-identified tumors were significantly less invasive than patient-identified tumors. Although age, family history, and tumor location played roles in the early detection of melanomas, the most important factor was diagnostic source. Thus, board-certified dermatologists play a key role in the early detection of malignant melanoma.

## 1. Introduction

Cutaneous malignant melanoma is a serious medical condition that represents a growing problem both in the United States and abroad [1]. Indeed, the increase in incidence of melanoma exceeds that of all other malignancies [2], and the cost of treating this disease is significant [3–6]. The increases in melanoma incidence rates are not simply due to increased public awareness but a real and growing threat to public health [7]. Thus, early detection is critical to minimizing melanoma severity and mortality, and important in reducing healthcare costs.

Efforts to better understand and improve early detection have focused on several areas of research, including skin-self examination (SSE) and public education [8–10], melanoma detection patterns [11–22], and risk factors [15, 21, 22].

Currently, in the United States, there is governmental ambivalence about recommending routine screening for skin cancer in asymptomatic patients in the primary care clinical setting using a whole-body skin examination [11]. However, recent evidence suggests that melanoma detection by a board-certified dermatologist is more effective than SSE and public education programs [12], and comparable to that of other screening tests, including mammography [13]. Moreover, in comparison with other physicians (e.g., internists, family practice physicians), melanomas detected by board-certified dermatologists are thinner, in an earlier stage of development, and have a significantly greater survival rate [12, 14–16]. This is consistent with evidence from surveys indicating that many general practitioners lack confidence in their ability to identify melanomas [17].

Thus, there is clear evidence that dermatologists are uniquely skilled in their ability to detect melanomas at an early stage, yet there is little data focusing on dermatologists as opposed to other physicians. Results of studies in the latter group indicate that clinicians initially identify between 14–25% of lesions found [16, 18–23]. These percentages are useful from an epidemiologic standpoint, but on a more practical level, this information is not tremendously helpful to a majority of dermatologists since none of these studies were performed in a private dermatology practice. Indeed, there has been only one study examining detection patterns in a private dermatology practice and they reported that 56% of lesions were initially identified by the dermatologist [12]. One finding that is consistent across all studies, however, is that lesions first identified by a physician are thinner than melanomas found by patients [12, 16, 19–23].

In contrast to pattern-of-detection studies, one of the least understood areas of research is the impact of a personal or family history of skin cancer on early detection. Effectiveness of skin cancer screening is thought to be increased if targeted to high-risk persons [24], however, results of studies are mixed [15, 21, 22]. It makes intuitive sense that patients with a family history of melanoma would be more vigilant in seeking out preventative measures, but given the paucity of relevant data, it is unclear whether this factor does play a major role in outcome.

Given these considerations, this study was designed/performed to address two important limitations of prior research, (1) a lack of information regarding patterns-of-detection as they relate specifically to dermatologists in a private dermatology clinic setting, and (2) the limited data related to the influence of personal and family history of skin cancer on those patterns and their influence on early detection. Thus, we examined whether melanomas self-identified by patients would be more invasive than those identified by a board-certified dermatologist at routine office screening visits, and that individuals with a personal or family history of skin cancer would be more likely to self diagnose a melanoma than those with no prior history.

## 2. Methods

We performed a retrospective chart review of patients (*N* = 201) diagnosed with melanoma by a board-certified dermatologist in a general dermatology clinic in Reno, Nevada, between 2002 and 2011. Seven subjects had either missing charts or a severe lack of data that warranted their exclusion from the study. Patients were initially identified using a computerized patient database, after which paper charts were used to obtain data including age, sex, whether subjects had a family history or personal history of melanoma or other skin cancer (e.g., basal cell carcinoma, squamous cell carcinoma), the location of the melanoma (i.e., head/neck, scalp, chest/trunk, back, arm/hand, leg/foot), Clark's level, Breslow's thickness at diagnosis (for advanced tumors), skin type, and their reaction to excessive sun exposure (i.e., burn and/or tan). Tumor severity was reclassified based on whether the melanoma was in situ, less than 0.75 mm, or greater than 0.75 mm in thickness. This variable was used in all analyses of tumor severity or invasiveness. In addition, documentation was reviewed to determine whether the patient identified the lesions themselves (or by a family member) or if they were first identified by the dermatologist during a routine office visit and screening skin exam. All diagnoses were confirmed through biopsy and read by an independent Board Certified Dermatopathologist. Beginning in 2006, dermoscopy was used as a supporting diagnostic tool by the clinician. Patients with invasive melanoma that were candidates for sentinel lymph node biopsy were referred to a regional melanoma center in San Francisco, CA. Statistical analyses were performed using the Statistical Analysis System (SAS Institute Inc, Cary, NC) on a PC computer. We first examined differences between DI and PI groups for age, gender, personal history of melanoma and/or other types of skin cancer, family history of melanoma and/or other types of skin cancer, lesion location, and tumor severity using t-tests and chi-square analyses. In order to correct for increased error rates from these multiple comparisons, a correction was applied to control for Type I errors (*P* < 0.01). A post-hoc logistic regression reporting odds ratios (OR's) was also performed to determine whether age, family history, personal history, location, or diagnostic source was predictive of tumor invasiveness while controlling for their potential interactions.

Institutional Review Board approval for this study was obtained from the University of Nevada, Reno, Office of Human Research Protection.

## 3. Results

The patients were men (*n* = 81) and women (*n* = 120) ranging in age from 7 to 91 (mean age was 60.3 ± 16.3 versus 54.6 ± 15.6, resp.). Ninety one percent (91%) of patients had a skin type of II, and 37% had a history of blistering sunburn. Over a 10-year period, *n* = 101, melanomas were initially identified by the dermatologist (DI group), with the rest initially identified by the patient or family members (PI group; *n* = 100) (Table 1). In three cases, patients were adopted and family history could not be determined, with the remaining subjects (*n* = 198) distributed among those with no family history (*n* = 121, 61%), a family history of melanoma (*n* = 30, 15%), and a family history of other types of skin cancer (*n* = 47, 24%). Personal histories were divided among subjects with no history (*n* = 89, 44%), and those with a history of melanoma (*n* = 18, 9%), atypical nevi (*n* = 20, 10%), and other types of skin cancer (*n* = 74, 37%). Overall, 158 (78.5%) subjects had melanoma in situ, 26 (13%) a Clark's Level II tumor, 12 (6%) a Clark's Level III, 4 (2%) a Level IV, and 1 (0.5%) a Level V tumor. The average Breslow's thickness for the 43 subjects in which it was measured was 0.73 mm. Twenty-seven subjects had a Breslow's thickness ≥0.75, and 16 subjects <0.75 (Table 2). The average Breslow thickness in the PI group was 0.79 mm (SD = 0.73 mm, *n* = 33) and 0.53 mm in the DI group (SD = 0.26 mm, *n* = 10).

Analysis of age yielded significant differences between the PI (mean age = 54.7 yrs, SD = 12.5) and DI groups (mean age = 60.7 yrs, SD = 16.7), (*t* = 2.9, *P* < 0.005), (Table 1). Diagnostic source was also significantly related to a family history of melanoma, with significantly more melanomas identified by patients when there was a family history of melanoma (DI = 7%, PI = 23%) compared to no family history (DI = 67%, *PI* = 55%), or a family history of other skin cancers (DI = 26%, PI = 22%),  (*χ* ^2^ = 9.7,  *P* < 0.01). No such association was found for a personal history of skin cancer (*χ* ^2^ = 9.5,  *P* < 0.05, n.s.). No between-group differences were found for gender, skin type, and history of blistering sunburns.

Differences in tumor types, severity, and location between DI and PI groups are provided in Table 2. There was a significant relationship between diagnostic source and tumor location with DI tumors more likely to be present on the chest/trunk (DI = 19%, PI = 14%), back (DI = 19%, PI = 14%), and legs (DI = 34%, PI = 21%), while PI tumors were more frequently identified on the face/neck (DI = 9%, PI = 29%) and scalp (DI = 1%, PI = 2%), (*χ* ^2^ = 15.4, *P* < 0.01). No between-group differences were found for gender, skin type, and history of blistering sunburns.

Analysis of diagnostic source and tumor severity resulted in significantly less invasive melanomas in the DI group compared to the PI group (*χ* ^2^ = 15.9, *P* < 0.0005). Examination of tumor type showed that for DI tumors, 90% were diagnosed as melanoma in situ, 7% were identified as Clark's level II, 2% level III, and 1% level IV. In contrast, PI tumors were 67% in situ, 19% Clark's level II, 10% level III, and 4% level IV.

The post-hoc logistic regression analysis revealed that only one significant variable predicted tumor invasiveness. Specifically, of all the predictor variables (age, family history, personal history, tumor location, and diagnostic source), only diagnostic source (i.e., PI versus DI) was a significant predictor of tumor invasiveness (OR = 0.19; 95% CI, 0.08–0.43; *P* < 0.0001).

## 4. Discussion

This is the first study to systematically examine the effects of family and personal history on detection patterns of cutaneous melanomas in a private dermatology clinic. Results of univariate analyses indicated that individuals with a family history of melanoma, but not other types of skin cancer, were significantly more likely to initially identify a melanoma themselves. No such finding was observed in individuals with a personal history of skin cancer and no gender effects were found, but age was related to detection patterns. Specifically, older patients were significantly more likely to have a negative family history and a dermatologist-identified melanoma. Also consistent with previous data, lesions identified by the dermatologist were more likely to occur on the chest, back, and legs as opposed to the face, neck, and scalp.

These results are consistent with previous studies demonstrating that dermatologist-identified cutaneous melanomas were significantly less severe than those identified by patients [12, 15, 16]. They also add to the relative paucity of data indicating that board-certified dermatologists identify roughly half of the lesions found during routine private dermatology clinic visits [12]. These results suggest that routine screening visits by a board-certified dermatologist in the private practice setting are crucial to detecting melanomas at an early stage. If patients wait to report suspicious lesions, melanomas are more likely to progress from local to regional or distant disease with a worse prognosis. All high-risk persons should see a physician for routine skin cancer screening exams throughout the year.

The relationship observed between a positive family history of melanoma and an increased likelihood of the patient self-identifying a melanoma is not surprising. We expected these high-risk individuals to be more aware and concerned about new skin growths resulting in greater vigilance with respect to skin changes. However, given the inconsistent findings in previous studies [15, 20–22], as well as the results of the logistic regression presented here, additional studies are warranted to determine the impact of this important factor within private dermatology clinic settings.

In contrast to family history, a past personal history of skin cancer including basal and squamous cell carcinoma, or melanoma, does not increase a person's odds of identifying a cancerous lesion. It makes intuitive sense that patients with a history of skin cancer are more likely to see a dermatologist for routine screening visits to ensure early diagnoses, while patients with no personal history are less likely to see a dermatologist regularly and only get screened after noticing a suspicious nevus.

It is worth noting that melanomas on the chest, trunk, and back were more likely to be found by a dermatologist. Previous studies of susceptibility suggest different etiologic pathways of melanoma development depending on the anatomical location [25]. It is possible that, given the etiological complexity, dermatologists are uniquely qualified among clinicians to identify lesions depending on a number of patient characteristics and the location of the lesion.

Finally, although age was not the primary variable of interest, results indicated that advanced age may be related to more advanced melanomas. Previous studies have identified age as an important prognostic factor of melanoma survival, independent of other factors like tumor thickness and ulceration [26]. These studies also suggest that age-related declines in immune-system functioning are the cause of the increased severity of melanomas found in older patients. Our finding that older patients with no family history of skin cancer were more likely to have a lesion identified by a dermatologist suggests that skin cancer education may be more effective if targeted to older individuals with no family history of melanoma.

Despite these findings, results of the logistic regression analysis cast some doubt on the significance of family history, age, and other variables on melanoma outcome. Specifically, when age, family history, personal history, tumor location, and diagnostic source were included simultaneously in the analysis, only the diagnostic source proved to be a significant predictor of melanoma invasiveness. Few studies have examined interactions among factors previously associated with tumor severity, and thus these results demonstrate that additional information is critical to understanding how these factors interact and eventually impact early detection. Most importantly, consistent with previous findings [12, 15, 16, 27], they suggest that board-certified dermatologists play a critical role in the early detection of malignant cutaneous melanomas.

Despite these findings, this study was limited given that it relied on a retrospective chart review from a single private dermatology practice. Thus, it lacked the scientific rigor of a controlled, prospective study. In order to increase the ability to generalize these findings across the population, additional studies in other regions, demographic groups, and clinical settings are warranted. However, despite these limitations, these results are consistent with previous studies and add to the growing literature indicating that melanoma screening by a board-certified dermatologist is associated with an earlier detection as compared to patients who self-identify lesions. Moreover, although not a significant finding, a family history of skin cancer may play a role in early detection.

## Figures and Tables

**Table 1:** Patient variables.

Variable	Patient-identified melanoma (*n* = 100)^a^		Dermatologist-identified melanoma (*n* = 101)^a^	
Age, Mean years (SD)	54.7 (16.7)		60.7 (12.5)	
Female gender	64		56	
Skin type (Fitzpatrick scale)	Type I	2.1	Type I	3
	Type II	92.6	Type II	91
	Type III	5.3	Type III	6
Positive history of blistering sunburns	39		43	
Personal history	None	54	None	34
	Melanoma	7	Melanoma	11
	Other^b^	39	Other^b^	55
Family history of melanoma	None	55	None	67
	Melanoma	23	Melanoma	7
	Other^b^	22	Other^b^	26

^a^Data are given as percentages unless otherwise indicated.

^b^Includes basal and/or squamous cell carcinoma and atypical nevus.

**Table 2:** Melanoma characteristics.

Variable	Patient-identified melanoma (*n* = 100)^a^		Dermatologist-identified melanoma (*n* = 101)^a^	
Breslow thickness	In-Situ	67	In-Situ	90
	<0.75 mm	20	<0.75 mm	7
	≥0.75 mm	13	≥0.75 mm	3

Clark's level	In-situ	67	In-situ	90
	Level II	19	Level II	7
	Level III	10	Level III	2
	Level IV	4	Level IV	1

Melanoma location	Face/Neck	29	Face/Neck	9
	Scalp	2	Scalp	1
	Chest/Trunk	14	Chest/Trunk	19
	Back	14	Back	19
	Arm/Hand	20	Arm/Hand	18
	Leg/Foot	21	Leg/Foot	34

^a^Data are given as percentages.
